# A convergent mechanism of sex determination in dioecious plants: Distinct sex-determining genes display converged regulation on floral B-class genes

**DOI:** 10.3389/fpls.2022.953445

**Published:** 2022-08-26

**Authors:** Xianzhi Zhang, Linsi Pan, Wei Guo, Yongquan Li, Wencai Wang

**Affiliations:** ^1^Department of Horticulture, College of Horticulture and Landscape Architecture, Zhongkai University of Agriculture and Engineering, Guangzhou, China; ^2^Department of Molecular of Biology, Science and Technology Innovation Center, Guangzhou University of Chinese Medicine, Guangzhou, China

**Keywords:** plant dioecy, sex determination, sex-determining genes, MADS-box genes, convergent mechanism

## Abstract

Sex determination in dioecious plants has been broadly and progressively studied with the blooming of genome sequencing and editing techniques. This provides us with a great opportunity to explore the evolution and genetic mechanisms underlining the sex-determining system in dioecious plants. In this study, comprehensively reviewing advances in sex-chromosomes, sex-determining genes, and floral MADS-box genes in dioecious plants, we proposed a convergent model that governs plant dioecy across divergent species using a cascade regulation pathway connecting sex-determining genes and MADS-box genes e.g., B-class genes. We believe that this convergent mechanism of sex determination in dioecious plants will shed light on our understanding of gene regulation and evolution of plant dioecy. Perspectives concerning the evolutionary pathway of plant dioecy are also suggested.

## Introduction

Dioecious plants have their male and female sexes individually separated. In the plant kingdom, just a small minority (5%−6%) of species are dioecious, distinct from the animal kingdom where gonochorism is common (Charlesworth, 2016). A large portion of these dioecious plants is commercially important including poplar (*Populus* spp.), kiwifruit (*Actinidia* spp.), garden asparagus (*Asparagus officinalis* L.), papaya (*Carica papaya* L.), spinach (*Spinacia oleracea* L.), hemp (*Cannabis sativa* L.), hardy rubber tree (*Eucommia ulmoides* Oliver), and pistachio (*Pistacia vera* L.). Notably, some widely-cultivated crops such as hexaploid persimmon (*Diospyros kaki* Thunb.), grape (*Vitis vinifera* L.), and strawberry (*Fragaria* × *ananassa* Duch.) are domesticated from their close dioecious relatives (Henry et al., 2018). Dioecy appears in various plant lineages, though less frequently. This provides us with a great opportunity to explore the potential convergent mechanisms in these plants (Leite Montalvão et al., 2021).

**Figure 1:**
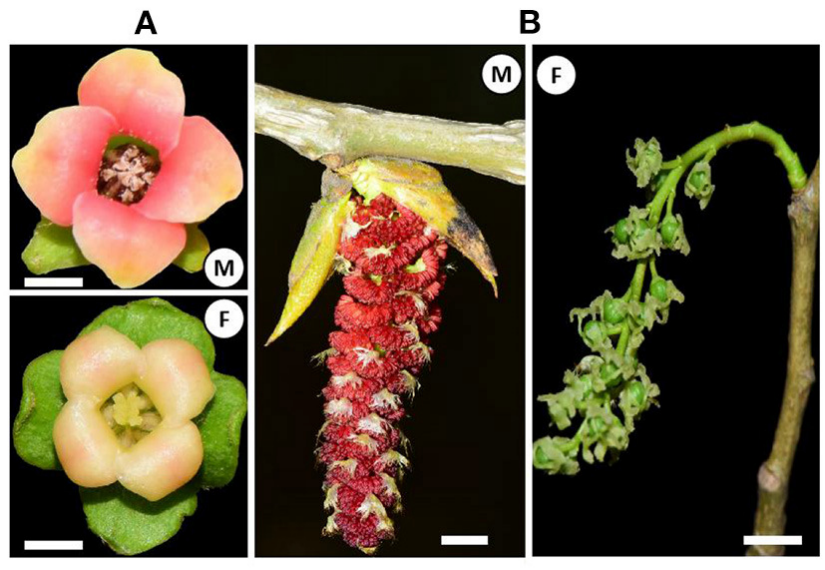
Unisexual flowers of *Diospyros lotus* (Type I flower) **(A)** and *Populus deltoides* (Type II flower) **(B)**. M, male flower; F, female flower. Scale bar = 1 cm.

Two morphological types of unisexual flowers have been recognized since the Darwinian era in dioecious plants (Darwin, 1877; Jabbour et al., 2022). The first one, the Type I flower, is unisexual by abortion, which is bisexual at its very early stages but becomes unisexual following the developmental arrest of stamen or carpel, e.g., *Diospyros lotus* L. (Figure 1A). The second one, the Type II flower, is unisexual from inception, with only the primordium of stamen or carpel at separate floral meristem (Mitchell and Diggle, 2005; Diggle et al., 2011), e.g., *Populus deltoids* cv. “Zhonghuahongye” (Figure 1B). Interestingly, Type I flowers are more frequently observed in dioecious plants in comparison with Type II flowers.

The rapid development of high-throughput sequencing techniques has enabled a full flourish of plant genomics. Sex-determining genes have been identified based on combined genomic and experimental approaches in certain dioecious plants with both Type I and II flowers (Carey et al., 2021; Renner and Müller, 2022). Taken together, these findings make it possible for us to elucidate the mechanism of sex determination in dioecious plants. Studies on poplar (Type II flower) (Leite Montalvão et al., 2022) and persimmon (Type I flower) (Yang et al., 2019) particularly indicate insightful clues on how the sex-determining genes are connected to the dioecious phenotype through the floral MADS-box genes. Impressively, although with distinct flowers, both studies revealed that the floral B-class genes are the downstream target of sex-determining genes (Theißen et al., 2016). This leads us to further explore the potential connection between the sex-determining genes and floral development genes and how they ultimately determine plant dioecy.

## Identification of the sex-determining region (SDR) on sex chromosomes

**Figure 2:**
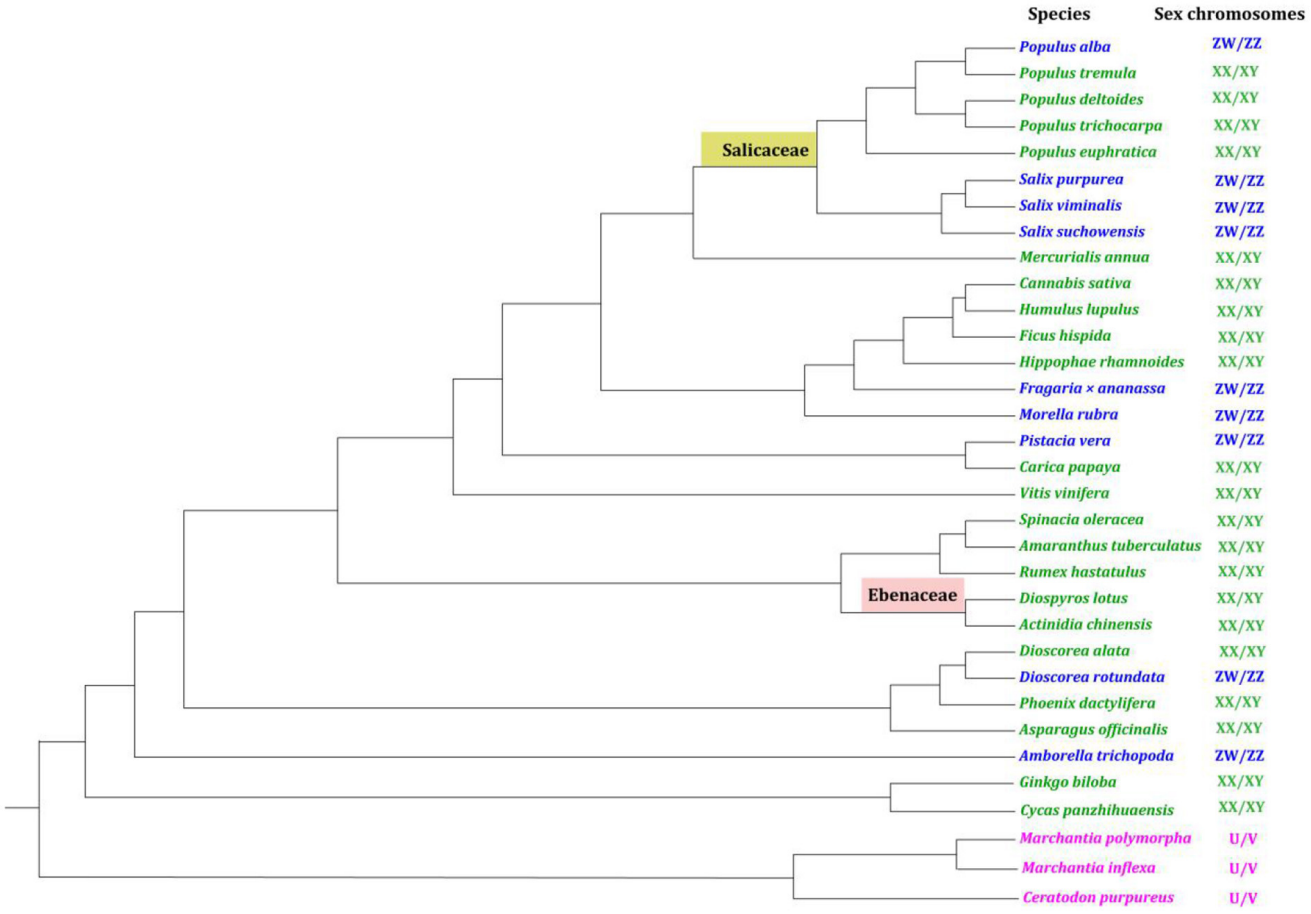
Dioecious plants with reference genomes and convinced sexual systems in a phylogenetic perspective. The male heterogametic system (XY/XX), female heterogametic system (ZW/ZZ), and haploid sex system (U/V) are shown in green, blue, and pink respectively. The distantly-related lineages of poplar (Salicaceae) and persimmon (Ebenaceae) are respectively highlighted in yellow and light red. The phylogenetic tree was reconstructed using chloroplast genome sequences *via* the online RAxML method in CIPRES (http://www.phylo.org/) (Supplementary Table S1).

Over fifty dioecious plant species have been sequenced since 2006 when the first dioecious genome (*Populus trichocarpa* Hook.) was published (Tuskan et al., 2006). But in these sequenced dioecious plants, only 33 species in 22 families (Supplementary Table S1) were found to have sex chromosomes (Ming et al., 2011; Baránková et al., 2020; Leite Montalvão et al., 2021; Charlesworth, 2022) to our best knowledge. The 33 dioecious plants with available genomes and sexual systems were summarized in a phylogenetic perspective (Figure 2, Supplementary Table S1). In general, three kinds of sex systems, i.e., male-heterogametic system, female-heterogametic system, and haploid sex system have been found in dioecious plants by far, corresponding to XX/XY, ZW/ZZ, and U/V sex chromosomes respectively. The number of species with XX/XY system (21) is greater than species with ZW/ZZ system (9) and U/V system (3) (Figure 2).

In the present genomic era, deep sequencing and high-quality assembly of sex chromosomes are undoubtedly critical to reliably identifying the non-recombining sex-determining region (SDR). There are three stages in deciphering SDR of dioecious plants that have been undertaken: (i) Traditional molecular techniques stage: genetic linkage mapping and bacterial artificial chromosome (BAC) were employed to overcome the encountered difficulties, for example, when assembling the Y chromosome of papaya in 2004 (Liu et al., 2004). However, these methodologies are either labor-intensive or hard to obtain the linkage map, particularly for species with long juvenile periods e.g., *E. ulmoides* (Wang et al., 2020). Moreover, extra small- or large-sized SDR, such as ~59 kb in *Morella rubra* Lour. (Jia et al., 2019) and ~50 Mb in *Ginkgo biloba* L. (Gong and Filatov, 2022) were also difficult to be reliably identified or orderly aligned based on genetic linkage mapping and BAC approaches. (ii) Short-read sequencing stage: The advent of high throughput sequencing techniques that generate an enormous amount of short reads (100–300 bp) boosted the rapid development of dioecious plant genomics. Although the output of the short sequencing reads is extremely high, for instance, Illumina HiSeq 4000 platform can generate up to >1 Tb per run, it remains problematic. On the one hand, the overall sequencing quality of short reads needs to be improved, more importantly, on the other, short reads fail to span certain genomic territories such as repetitive regions (e.g., transposable elements and tandem repeats) which would normally accumulate on SDR of the sex chromosomes (Li et al., 2016; Muyle et al., 2017). It is thus understandable that both X and Y (or Z and W) sex chromosomes were either largely absent or partially assembled as a chimera in some of the published dioecious genomes (Supplementary Table S1 and Figure 2). (iii) Long-read sequencing stage: Long-read sequencing techniques including Nanopore, PacBio, and Hi-C chromatin conformation techniques that produce long or ultra-long reads (20–100 kb) largely remedied the deficiency given by short-read sequencing, which can bridge up to 15–30 kb genomic territories (Carey et al., 2021). Contigs of X and Y chromosomes of *Ficus hispida* L. were primarily assembled using Pacbio long reads (~20 kb) before the fineX (21.9 Mb) and Y (22.6 Mb) chromosomes were fully constructed with Hi-C reads (500–700 bp) (Zhang et al., 2020). The SDR of *Cycas panzhihuaensis* L. Zhou and S. Y. Yang were configured based on Nanopore long-reads and Hi-C data, giving rise to a 45.5 Mb-sized Y haplotype (Liu et al., 2022).

Many of the genome-sequenced dioecious plants have only one sex, i.e., either male or female, selected for sequencing due to technique or budget issues leaving their sex chromosomes not covered by genomic data (Baránková et al., 2020) though experimental data may be available. Nevertheless, we need to be open to the varied sequencing techniques and/or assembly methodologies to tackle practical problems in genomics studies of dioecious plants. A T2T gapless human X chromosome (Miga et al., 2020) and highly continuous human Y chromosome (Kuderna et al., 2019) have been newly constructed by long-read sequencing data. This may be also the bright direction to decipher the intractable sex chromosomes of dioecious plant species in the near future. In particular, deciphering the haplotype-phased genome of a heterogametic individual, such as the male with XY sex chromosomes in the male-heterogametic system and/or the female with ZW sex chromosomes in the female-heterogametic system, will accelerate the identification of sex-determining genes in dioecious plants.

## Divergent sex-determining genes

### Two-gene model

The “two-gene model” (Westergaard, 1958; Charlesworth and Charlesworth, 1978) was first proposed to explain the evolution of dioecy from a hermaphrodite ancestor. In this model, a recessive male-sterility mutation in the male-promoting factor (M) initially results in a gynodioecious population i.e., co-existence of females and hermaphrodites. Then, a dominant female sterility mutation occurs in the suppressor of femaleness (SuF). Both M and SuF locate at the SDR, leading to dioecy. This model was confirmed by practical evidence found in kiwifruit (*Actinidia* spp.) and garden asparagus (*Actinidia officinalis*) (Table 1). Most species of *Actinidia* are dioecious, among which *Actinidia chinensis* Planch is a worldwide well-known fruit crop with XX/XY chromosomes (Wang et al., 2016). It has a small-sized (~0.8 Mb) SDR housing two male-specific expressed genes, i.e., *SyGI* and *FrBy* (Akagi et al., 2019). Gene *SyGI* (“*Shy Girl*”) is a type-C cytokinin response regulator. Transgenic expression of *SyGI* in *Arabidopsis thaliana* (L.) Heynh. and *Nicotiana tabacum* L. resulted in suppression of carpel development but no effect on stamen (Akagi et al., 2018). Recent work using the CRISPR/Cas9 gene editing technique to mutagenize *SyGl* in *A. chinensis* male plants gave rise to hermaphrodites with functional gynoecium and androecium, validating its identity of SuF (Varkonyi-Gasic et al., 2021). The second gene *FrBy* (*Friendly Boy*) is critical for viable pollens formation, functioning as an M factor. Transgenic expression of *FrBy* converted *A. chinensis* females into hermaphroditic individuals (Akagi et al., 2019).

**Table 1:** Sex-determining genes and/or strong candidates identified in dioecious plants.

**Type of unisexual flowers**	**Species (family)**	**Sex system**	**Sex-determining genes and function**	**References**
Type I flowers: Unisexual by abortion	*Actinidia* spp. ^*^(Actinidiaceae)	XX/XY	*SyGI*: Female suppressor *FrBy*: Male activator	Akagi et al. (2018, 2019)
	*Asparagus officinalis* ^*^(Asparagaceae)	XX/XY	*SOFF*: Female suppressor *aspTDF1*: Male activator	Harkess et al. (2017, 2020)
	*Carica papaya* (Caricaceae)	XX/XY	*CpSVP*: MADS-box gene, unknown function *CpSERK* and *CpCAF1AL*, unknown function	Urasaki et al. (2012), Lee et al. (2018)
	*Diospyros lotus* ^*^(Ebenaceae)	XX/XY	*OGI*: Male activator	Akagi et al. (2014, 2020)
	*Ficus hispida* (Moraceae)	XX/XY	*FhAG2*: MADS-box gene, putative male activator	Zhang et al. (2020)
	*Fragaria virginiana F. chiloensis* (Rosaceae)	ZW/ZZ	*GMEW*: unknown function *RPP0W*: unknown function	Tennessen et al. (2018)
	*Nepenthes gracilis N. rafflesiana N. pervillei* (Nepenthaceae)	XX/XY	*DYT1*: Putative male activator *SEP1*: MADS-box gene, unknown function	Scharmann et al. (2019)
	*Phoenix* spp. (Arecaceae)	XX/XY	*CYP703* and *GPAT3*: Putative male activator *LOG-like*: Putative female suppressor	Torres et al. (2018)
	*Silene latifolia* (Caryophyllaceae)	XX/XY	*SlAP3*: MADS-box gene, unknown function *SlSTM*: unknown function *SlCUC*: unknown function	Zluvova et al. (2006); Cegan et al. (2010)
	*Vitis vinifera* ^*^ (Vitaceae)	XX/XY	*VviINP1*: Putative male activator *VviPLATZ1*: Female activator	Massonnet et al. (2020), Iocco-Corena et al. (2021)
Type II flowers: Unisexual from inception	*Cycas panzhihuaensis* (Cycadaceae)	XX/XY	*CYCAS_034085*: MADS-box gene, unknown function	Liu et al. (2022)
	*Ginkgo biloba* (Ginkgoaceae)	XX/XY	*GbMADS18*: MADS-box gene, unknown function *Gb_15883*: unknown function *Gb_15884*: unknown function *Gb_15885*: unknown function *Gb_15886*: unknown function *Gb_28587*: MADS-box gene, unknown function	Zhang et al. (2019); Liao et al. (2020)
	*Morella rubra* (Myricaceae)	ZW/ZZ	*MrCPS2*: unknown function *MrASP2*: unknown function	Jia et al. (2019)
	Populus alba^*^ (Salicaceae)	ZW/ZZ	*ARR17*: Female activator	Müller et al. (2020)
	Populus tremula^*^P. trichocarpa^*^ (Salicaceae)	XX/XY	*ΨARR17-IR :* Female suppressor	Müller et al. (2020)
	Populus deltoids^*^ (Salicaceae)	XX/XY	*FERR-R*: Female suppressor *MSL*: Male activator	Xue et al. (2020)
	*Populus euphratica* (Salicaceae)	XX/XY	*ΨRR-IR*: Putative female suppressor	Yang et al. (2021)
	*Salix purpurea* (Salicaceae)	ZW/ZZ	*SpRR9*: Putative female activator	Zhou et al. (2020a)
	*Spinacia oleracea* (Amaranthaceae)	XX/XY	*NRT1/PTR6.4*: Putative male activator	Ma et al. (2022)

* Indicates the sex-determining genes of these species have been functionally studied.

Garden asparagus (*A. officinalis*, Asparagaceae) also has XX/XY sex chromosomes and a similar small-sized SDR (~1 Mb) as seen in kiwifruit (Harkess et al., 2017). Two SDR-encoded genes, i.e., *SOFF* (*Suppressor of Female Function*) and *aspTDF1* (*Defective in Tapetum Development and Function 1*) expressed specifically in males (Harkess et al., 2020). Knockout of *SOFF* by gamma irradiation converted males into hermaphrodites. Additionally, mutagenesis of *aspTDF1* in males by Ethyl Methanesulfonate (EMS) resulted in asexual neuters where neither carpels nor stamens developed normally. When both *SOFF* and *aspTDF1* genes underwent knockout, males directly changed into females. All these findings provided robust evidence that the two Y-encoded genes *SOFF* (SuF factor) and *aspTDF1* (M factor) were physically linked at SDR to co-determine the sex of the garden asparagus. Most notably, the male-promoting genes in kiwifruit (*FrBy*) and asparagus (*aspTDF1*) both function in tapetum for male flower fertility (Andreuzza, 2020), suggesting a convergent mechanism.

No direct experimental data were available for the identified sex-determining candidates in grape (*V. vinifera*) and date palm (*Phoenix dactylifera* L.), but the “two-gene model” can most likely be applied to them (Table 1). In grape, two SDR-encoded genes might be potential sex-determining genes: *VviINP1* likely participated in pollen fertility and acted as an M factor (Massonnet et al., 2020; Zou et al., 2021), and *VviPLATZ1* could suppress carpel development and most likely functioned as SuF factor (Iocco-Corena et al., 2021). In 13 *Phoenix* species, three genes (*CYP703, GPAT3*, and *LOG-like*) that were specifically expressed in male flowers were determined as strong sex-determining candidates (Torres et al., 2018). *CYP703* and *GPAT3* might affect pollen development while *LOG-like* genes likely suppressed carpel development (Torres et al., 2018).

### One-gene model

In contrast, sex determination of several other dioecious plants (Table 1) is known to follow the “one-gene model” that was firstly reported in persimmon (*Diospyros*, Ebenaceae) (Akagi et al., 2014; Charlesworth, 2016; Renner and Müller, 2022). In this model, one single gene located at SDR is critical to determine the sex *via* an epistatic gene interaction, rather than physical linkage with downstream genes. The SDR (~1 Mb) of Caucasian persimmon (*D. lotus*) contained one pseudogene *OGI* (*Oppressor of MeGI*) which exhibited male-specific expression. The *OGI* had an autosome-located duplicate gene, *MeGI* (*Male Growth Inhibitor*) which showed female-biased expression in floral buds. Importantly, *OGI* housed two identical inverted repeat sequences (IRs) which generated hairpin RNAs and 21-nt small RNAs (sRNAs) to return silence *MeGI via* RNA interference (RNAi) (Akagi et al., 2014, 2016, 2020; Akagi and Charlesworth, 2019). *MeGI* could repress androecium development, causing femaleness in transgenic *A. thaliana, Nicotiana benthamiana* Domin, and *N*. *tabacum*. When *OGI* was introduced in the *MeGI* transgenic plants, male sterility was restored. These results demonstrated that *OGI* functioned alone as the sex-determining gene in persimmon (Akagi et al., 2014; Akagi and Charlesworth, 2019).

Nearly all the *Populus* species in Salicaceae are dioecious (Yang et al., 2021) possessing both XX/XY and ZW/ZZ sexual systems, which makes it a model group for the study of plant sex determination (Cronk and Müller, 2020). Geraldes and colleagues (2015) (Geraldes et al., 2015) firstly performed whole-genome re-sequencing of *P. trichocarpa* and conducted a genome-wide association study (GWAS). They consequently revealed an XX/XY system presence in species belonging to both section Tacamahaca (*P. trichocarpa* and *Populus balsamifera* L.) and Aigeiros (*Populus deltoids* Marshall and *Populus nigra* L.). A small-sized (~100 Kb) SDR was identified in the Y chromosome (Chr. 19) of *P. trichocarpa*. It contained 12 protein-coding genes, including *A. thaliana* orthologs *ARR17* (*Arabidopsis Response Regulator 17*) and *MET1* (*Methyltransferase 1*). Subsequent studies on *P. balsamifera* further revealed that *ARR17* probably played a direct role in poplar sex determination (Bräutigam et al., 2017; Sanderson et al., 2019; Cronk et al., 2020; Zhou et al., 2020b). These aforementioned studies however lacked experimental data to evidence the biological function of *ARR17* in *Populus*. Only recently, Müller et al. (2020) demonstrated the feminizing function of *ARR17* by CRISPR/Cas9-induced mutations in *Populus tremula* L. (Müller et al., 2020). *ARR17* in *Populus* is also a cytokinin-related gene, similar to the above-mentioned *MeGI* gene of *Diospyros* (Akagi et al., 2020). CRISPR/Cas9 knockout of *ARR17* converted female *P. tremula* into males but made the males unchanged (Müller et al., 2020). Another group (Xue et al., 2020) independently performed the transgenic analyses of *ARR17* from *P. deltoids* (here named *FERR*) (Xue et al., 2020). They revealed stigma exaggeration and carpel-like sepal phenotypes in transgenic plants of *A. thaliana*, confirming the feminizing function of *ARR17* (Xue et al., 2020). Interestingly, the *ARR17* gene itself was found in the pseudo autosomal region of *P. deltoids* and *P. tremula*, but not in the SDR (Müller et al., 2020; Xue et al., 2020). Instead, a Y-specific pseudogene of *ARR17* (called Ψ*ARR17-IR*) was seated at the SDR of *P. deltoids* and *P. tremula* (Müller et al., 2020; Xue et al., 2020). Significantly, as a partial duplicate of *ARR17*, Ψ*ARR17-IR* could produce 24-nt sRNAs to silence *ARR17* reversely through RNA-directed DNA methylation (RdDM) (Müller et al., 2020; Xue et al., 2020).

The “one gene model” has been applied to several other dioecious plants beyond *Diospyros* and *Populus* (Table 1). For example, *Salix purpurea* L. (Zhou et al., 2020a) and *Salix viminalis* L. (Almeida et al., 2020) carried homologs of *ARR17* (*SpRR9*) at the SDR of the W chromosome (Zhou et al., 2020a). *F. hispida* had an SDR-located *FhAG2* gene which was identified as the putative sex-determining gene (Zhang et al., 2020). *Spinacia oleracea* contained a Y-specific gene *NRT1/PTR6.4* that was considered to be a strong candidate for sex determination (Ma et al., 2022). More recently the gymnosperm species *C. panzhihuaensis* was reported to have one SDR-located sex-determining candidate *CYCAS_034085* (Liu et al., 2022). It has been proposed that the evolution of dioecy from monoecy likely preferred the flexible “one-gene model,” whereas that from hermaphrodite might be accomplished by the “two-gene model” (Cronk, 2022). Regardless of the number of genes, both one- and two-gene models include the genetic connection of two independent genes. The difference rests on that whether the two sex-determining genes are physically linked together at the SDR of the Y (or W) chromosome (two-gene model), as *FrBy*/*SyGl* in kiwifruit (Akagi et al., 2019) and *SOFF*/*aspTDF1* in garden asparagus (Harkess et al., 2020), or the SDR-located sex-determining gene epistatically regulates its (pseudo)autosome-located homologous counterpart (one-gene model), as *OGI*/*MeGI* in persimmon (Akagi et al., 2014, 2020; Akagi and Charlesworth, 2019) and Ψ*ARR17-IR*/*ARR17* in poplar (Müller et al., 2020; Xue et al., 2020; Yang et al., 2021).

## Floral MADS-box genes regulated by sex-determining genes

**Figure 3:**
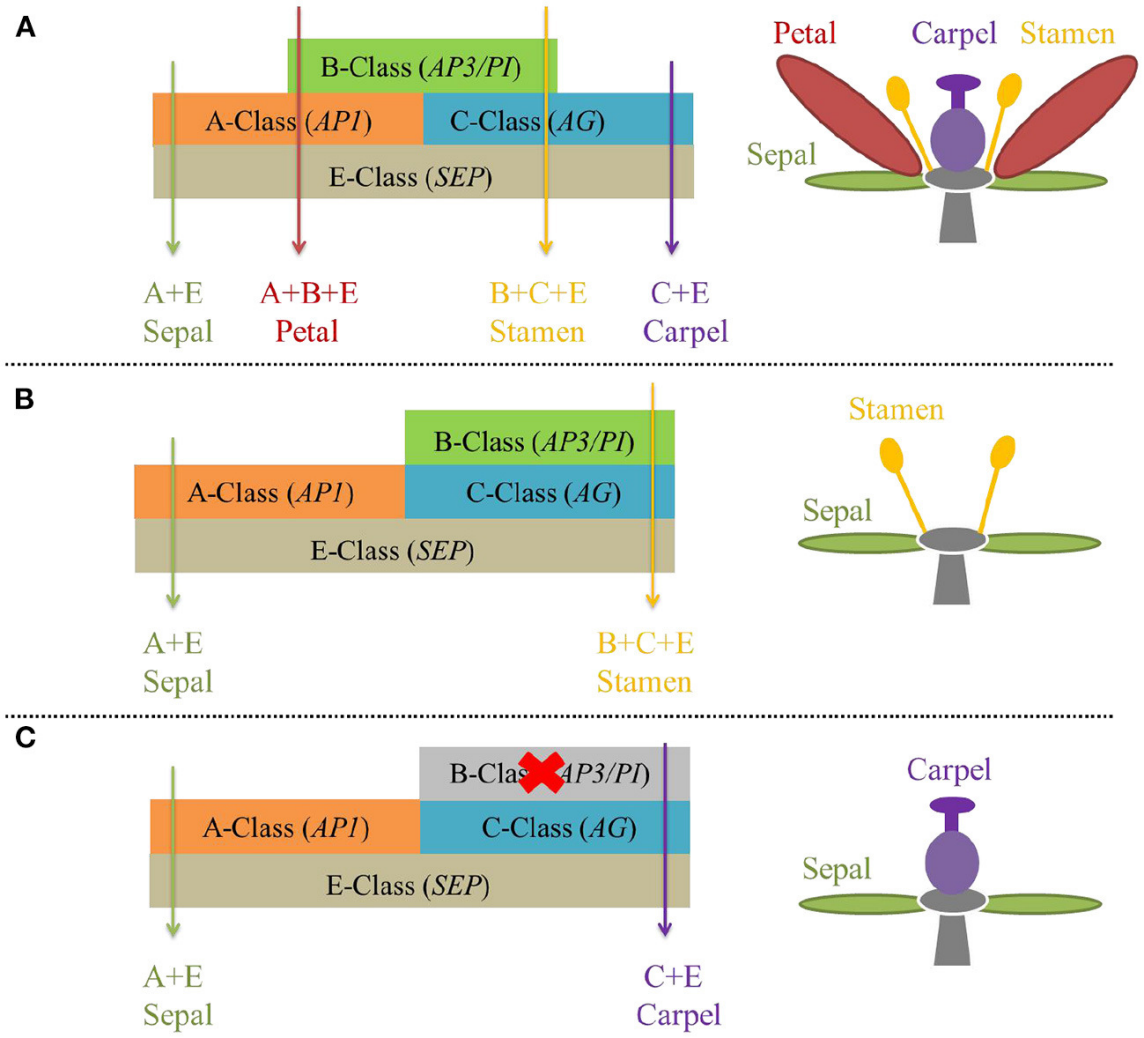
The “ABCDE model” of flower development, with D genes that specify ovule unshown. **(A)** B-class gene's normal expression makes perfect flowers. **(B)** Shifted and expanded expression domain of B-class genes permits male flowers. **(C)** Repression of B-class genes gives rise to female flowers.

Floral MADS-box genes are well known for their significant role in flower development according to the extended ABCDE model of perfect flower development (Theißen, 2001). The four whorls of perfect flowers from outside to inside i.e., sepal, petal, stamen, and carpel are specified by class A + E, A + B + E, B + C + E, and C + E genes respectively in the ABCDE model (Figure 3A) (Theißen, 2001). The frequently seen A-class gene *AP1* (*APETALA1*), B-class genes *AP3* (*APETALA3*) and *PI* (*PISTILLATA*), and C-class gene *AG* (*AGAMOUS*) were first discovered in *A. thaliana*, the model plant (Theißen et al., 2016). Notably, the spatial expression domains of the floral MADS-box genes are flexible (Soltis et al., 2007). For example, the expression of B-class genes has been expanded to whorl-1 i.e., covering the A-class domain in basal eudicot *Aquilegia* (Kramer et al., 2007) and monocot Liliaceae (Kanno et al., 2003), leading to petal-like sepals. The B-class gene *QsPI* was exclusively expressed in male flowers of *Quercus suber* L. and *QsPI* transgene in *pi-1* mutant *A. thaliana* successfully rescued the stamen function (Sobral and Costa, 2017). Therefore, when the expression domain of B- and C-class genes are overlapped, unisexual flowers e.g., male (Figure 3B) or female (Figure 3C) develop *via* switching on or off B-class genes (*AP3* and *PI*) (Soltis et al., 2007; Shan et al., 2019; Jabbour et al., 2022).

Floral MADS-box genes played key roles in the unisexual flower development of dioecious plants (Jabbour et al., 2022). For instance, B-class genes *SpAP3* and *SpPI* were found to express specifically in male floral primordial of *S. oleracea*, but absent in female flowers (Pfent et al., 2005). Subsequent functional analysis of B- (*SpAP3*/*SpPI*) and C-class (*SpAG*) genes documented that suppression of B-class expression converted male flowers into a female, indicating that regulation of B-class genes was associated with dioecy establishment in spinach (Sather et al., 2010). Similarly, B-class genes (*ThdAP3-2a/b*) of *Thalictrum dioicum* L. (Ranunculaceae) were known to be only expressed in male flowers but not in any stage of female flowers (Di Stilio et al., 2005). Besides, our group initially found one B-class gene, *EuAP3*, with male-biased expression in *E. ulmoides* (Wang and Zhang, 2017), which was confirmed by a different group working on the floral development of *E. ulmoides* (Zhu, 2019). This indicates that B-class genes were probable key sex regulatory factors of *E. ulmoides* (Wang et al., 2018, 2020). Moreover, an intact floral B-class gene *SlAP3Y* in *Silene latifolia* Poir. was found to be Y-specific and had an autosomal paralog *SlAP3A* (Matsunaga et al., 2003). Expression analysis revealed that *SlAP3Y* was highly expressed in stamens, but *SlAP3A* was specifically expressed in petals. This suggested that the B-class genes themselves might reside at the SDR of *S. latifolia* and act as masculinizing factors.

Recently, the regulatory pathway that bridges sex-determining genes and floral MADS-box genes were uncovered in *Populus* (Salicaceae) and *Diospyros* (Ebenaceae). In *P. tremula*, B-class gene *PI* was highly expressed in the *arr17* CRISPR mutants (males), in contrast to its low expression level in the isogenic females (*ARR17*) (Leite Montalvão et al., 2022). Notably, the meristem identity gene *UFO* (*Unusual Floral Organs*) was simultaneously and highly derepressed in *arr17* mutants. On the other hand, *UFO* encoded an F-box protein as a transcriptional cofactor with *LFY* (*LEAFY*) to activate the expression of B-class genes (Ng and Yanofsky, 2001; Theißen et al., 2016). Accordingly, it is probable that the genetic cascade of (Ψ*ARR17-IR*)-*ARR17*-*UFO*-*PI* controlled the sex of *P. tremula* (Leite Montalvão et al., 2022). In females, the normally expressed *ARR17* gene repressed downstream *UFO*-*PI* module, giving rise to female flowers; whereas in males, Y-specific Ψ*ARR17-IR* generated a large number of sRNAs to silence *ARR17*, restoring the expression of *UFO*-*PI* to produce male flowers (Leite Montalvão et al., 2022). Interestingly, the female-biased expression of *ARR17* was consistently correlated with the male-biased expression of *AP3* and *PI* in *P. balsamifera* (Cronk et al., 2020), suggesting a conserved sex-determination network in poplar.

Likewise, in persimmon, the feminizing gene *MeGI* also negatively regulated the B-class gene *PI* (Yang et al., 2019). *MeGI* directly controlled the expression of *SVP* (*Short Vegetative Phase*) which is an upstream repressor of floral B- and C-class genes (Theißen et al., 2016). In diploid dioecious *D. lotus*, the stable expression of *OGI* caused accumulation of sRNAs which in return silenced *MeGI*, so the B-class genes could normally express to produce male flowers. On the contrary, when B-class genes were repressed by the upstream *MeGI*-*SVP* module, only female flowers developed (Yang et al., 2019). Additionally, the *OGI*-*MeGI*-*SVP*-*PI* regulation cascade was applicable in monoecious hexaploid persimmon (*D. kaki*). In *D. kaki*, a 268-bp SINE-like element inserted into the promoter region of the *OGI* gene repressed its expression and subsequently produced female flowers (Akagi et al., 2016). In short, the aforementioned studies give a broad hint that the B-class genes play significant roles in determining the sex of dioecious plants.

## A convergent model underlying sex determination

Although a total of 21 and 16 sex-determining genes/candidates were identified in species with Type I and II flowers respectively (Table 1 and Figure 1), consensus gene sequences were lacking between phylogenetically divergent species that either share the same or have different flower types. Remarkably it seems that those divergent genes are functionally convergent, for example, a group of male-promoting e.g., *FrBy* in kiwifruit (Varkonyi-Gasic et al., 2021), *aspTDF1* in garden asparagus (Harkess et al., 2020), *CYP703* and *GPAT3* in date palm (Torres et al., 2018), *VviINP1* in grape (Massonnet et al., 2020), and *DYT1* in nepenthes (Scharmann et al., 2019) are all involved in the pollen development. This is indicative of a potential convergent mechanism governing the sex determination of dioecious plants.

**Figure 4:**
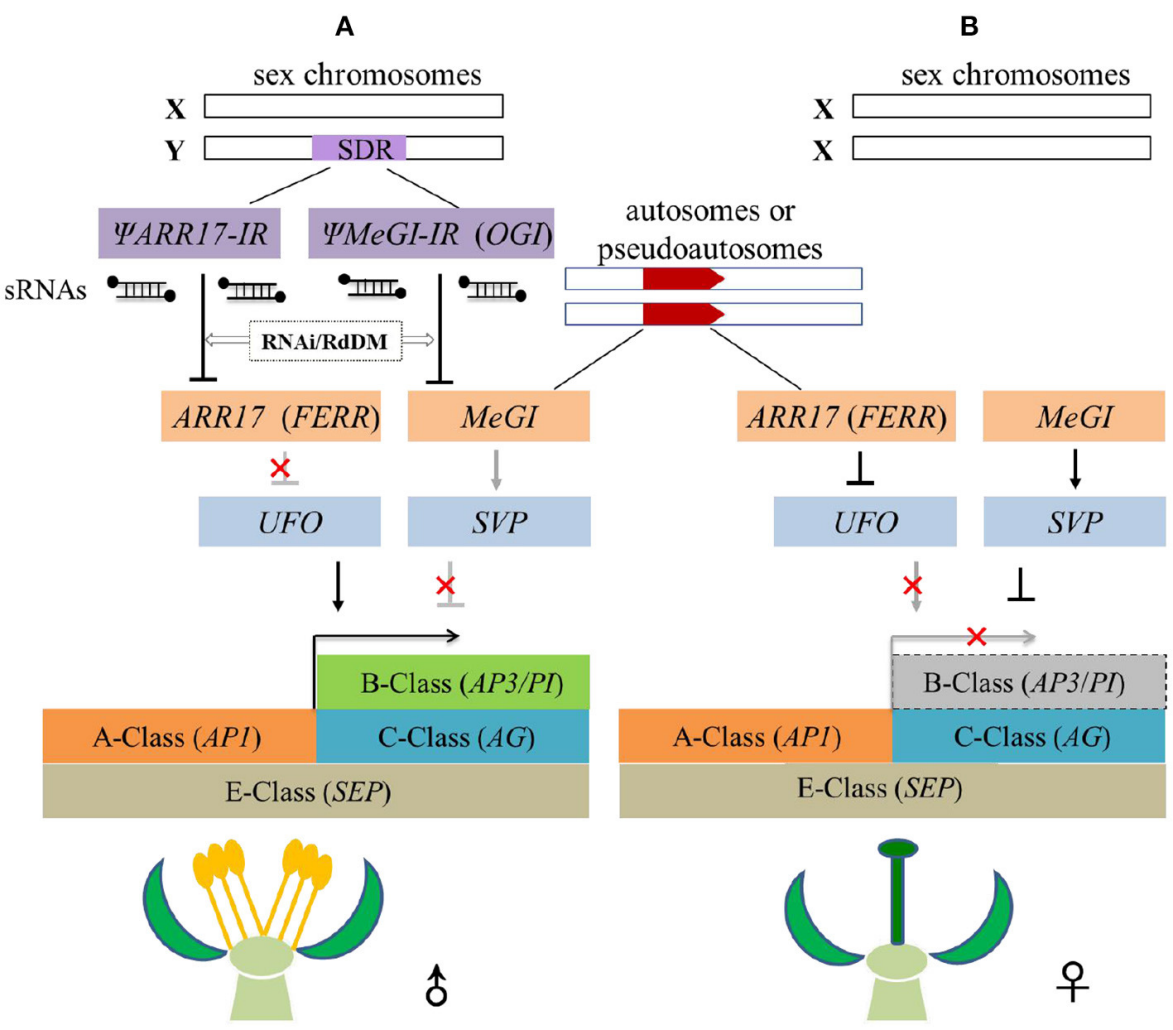
Convergent model of sex-regulation cascades from upstream sex-determining genes to downstream B-class genes in dioecious plants. **(A)** Duplicated pseudogenes (Ψ*ARR17-IR* or Ψ*MeGI-IR*) in the SDR of the Y chromosome generate sRNAs that dominantly repress their counterpart feminizing factors (*ARR17* or *MeGI*) on autosomes/pseudo autosomes *via* epigenetic RNAi/RdDM pathway. Downstream B-class genes are activated either by the expression of activator (*UFO*) or the suppression of repressor (*SVP*), producing only male flowers. **(B)** In XX individual, no pseudogenes of feminizing factors are available, repressor (*SVP*) of B-class genes are thus expressed, or activator (*UFO*) is repressed, and the expression of B-class genes are suppressed forming only female flowers.

Persimmon (Ebenaceae) and poplar (Salicaceae) diverged ~150 million years ago and possess distinct unisexual flowers as described previously (Figure 1) (Diggle et al., 2011; Li et al., 2019), but they share similar sex-regulation mechanisms. On the one hand, sex determination systems in *Populus* and *Diospyros* both support the “one-gene model,” both involving a non-SDR-located sex-switch regulator (*ARR17* in *Populus, MeGI* in *Diospyros*) that is toggled on off by its SDR-located pseudogene (Ψ*ARR17-IR* in *Populus, OGI* in *Diospyros*) on the Y chromosome. On the other hand, in both lineages, sex-determining genes ultimately function on B-class genes forming a regulatory cascade, i.e., (Ψ*ARR17-IR*)-*ARR17*-*UFO*-*PI* in *Populus* and *OGI*-*MeGI*-*SVP*-*PI* in *Diospyros*. Moreover, earlier work on *S. latifolia* (Matsunaga et al., 2003), *S. oleracea* (Pfent et al., 2005; Sather et al., 2010), *T. dioicum* (Di Stilio et al., 2005), and our current work on *E. ulmoides* (Wang and Zhang, 2017; Wang et al., 2018, 2020) all suggest the possibility of a universal convergent model that connects upstream sex-determining genes with downstream dioecious phenotypes (Figure 4). In this model, the spatial expression domain of B-class genes (*AP3*/*PI*) overlaps with the C-class gene (*AG*) and ultimately gets fixed at the population level. Only male flowers develop when B-class genes are normally expressed (Figure 4A), and only female flowers appear if B-class genes are repressed (Figure 4B).

Two steps are indispensable for the evolution of dioecy in this model (Figure 4). First, a feminizing mutation happens and gets fixed in the ancestral co-sexual (hermaphrodite or monoecy) populations. This feminizing mutation most likely occurs in the upstream negative regulators of B-class genes (*AP3*/*PI*), such as the *ARR17* in poplar (Müller et al., 2020) or *MeGI* in persimmon (Akagi et al., 2014). The interaction between feminized factors and B-class genes may be indirect *via* intermediate processes, for example through floral meristem genes *UFO* (Leite Montalvão et al., 2022) and *SVP* (Yang et al., 2019). Second, an SDR-located masculinizing mutation arises to repress the feminized genes and co-segregates with sex. This masculinizing mutation probably occurs in paralogs of the feminized factors, such as the *ΨARR17-IR* in *P. tremula* and *P. deltoids* (Müller et al., 2020; Xue et al., 2020), and *OGI* (here in this model referred to as *ΨMeGI-IR*) in *D. lotus* (Akagi et al., 2020). The masculinized gene thus could silence its paralogous feminized gene *in trans via* epigenetic regulations e.g., RNAi or RdDM (Akagi et al., 2014, 2020; Müller et al., 2020; Xue et al., 2020). These two mutations eventually achieve to determine the floral sexuality by toggling on/off the expression of B class genes, resulting in dioecy (Figure 4).

Speculatively, this model could be further extended to dioecious species that follow the “one-gene model.” Recent genomics studies on spinach (Ma et al., 2022), cycad (Liu et al., 2022), and Banyan tree (Zhang et al., 2020) all identified only one SDR-located gene that showed a male-specific expression pattern (Table 1). Therefore, all these species probably follow the convergent model we proposed here. Indeed, the sex determination mechanism *via* a single SDR-located gene is relatively more flexible and efficient compared to the classical “two-gene model” (Cronk and Müller, 2020; Renner and Müller, 2021). For instance, the “one-gene model” explained well the phenomena of sexual system transition between XY/XX (*P. tremula*) and ZZ/ZW (*P. alba*) in a simple way (Müller et al., 2020). The plant “one-gene model” was usually thought of as reminiscent of the human sex-determining system that has *SRY* (*Sex-Determining Region Y*) gene acting alone as a sex determinant (Sinclair et al., 1990; Graves, 2016). This convergent model thus resembles the bottom-up regulatory model of animal sex determination (Feng et al., 2020). Both models suggested that the sex-determining genes might be divergent across different species, but the regulatory cascades eventually converged to a conserved downstream hub that directly affects the development of reproductive organs. The floral B-class genes are most likely the conserved hub to control the unisexual flower development and sex of the individual (Figure 4).

Additionally, this convergent model itself can be varied. First, the feminization gene *per se* may locate at SDR and co-segregate with sex. This is the case of *P. alba* which elided the top module of epigenetic regulation (Ψ*ARR17-IR*/*ARR17*) (Müller et al., 2020). In the next place, B-class genes (*AP3*/*PI*) may be directly regulated by the feminized genes, omitting the middle module of activators (e.g., *UFO* in poplar) or repressors (e.g., *SVP* in persimmon). Yet this possibility remains to be further verified. And third, plant sex determination may also affect other important traits in addition to floral characteristics (Sanderson et al., 2019). For example, the flower odor-compounds of *S. latifolia* (Zluvova et al., 2010) and the leaf rubber content of *E. ulmoides* (Wang and Zhang, 2017) differed significantly between the sexes. To our knowledge, the MADS-box gene family contains several gene members, e.g., *c*.100 in *E. ulmoides* (Wang and Zhang, 2017), that function fundamentally and broadly in plant growth and development beyond flower development (Theißen, 2001; Soltis et al., 2007). Therefore, it is logical to speculate that different MADS-box gene members that influence important traits or phenotypes could be regulated by sex-determining genes as well.

## Conclusion and outlook

Dioecy has independently evolved more than hundreds of times across various plant lineages (Henry et al., 2018). Multiple-omics studies on dioecious plants accelerate the discovery of sex-determining genes, supporting both the “two-gene model” and “one-gene model” (Charlesworth, 2016; Renner, 2016). Recent findings from poplar (Leite Montalvão et al., 2022) and persimmon (Yang et al., 2019), as well as studies in other dioecious species (Wang and Zhang, 2017; Zhang et al., 2020; Liu et al., 2022; Ma et al., 2022), led us to propose a convergent model of plant sex determination. According to this model, the sex-determining genes may vary across divergent species, but they eventually converged into floral MADS-box genes, B-class genes, in particular, to determine sexuality.

Despite rapid advances in sex determination in dioecious plants, more issues need to be addressed. On the one hand, to determine SDR on sex chromosomes and identify the SDR-located sex-determining genes should be performed in a broad range, e.g., *via* involving distantly-related dioecious species or incorporating certain dioecious plants and their close non-dioecious relatives. This would assist us in better understanding the evolution of the sex determination mechanism. On the other hand, genome-wide identification and functional analysis of the MADS-box genes in dioecious species are encouraged to be conducted. Novel molecular techniques e.g., long-read sequencing in combination with genome-editing approaches will extensively facilitate sex chromosome assembly and sex-determining genes identification (Masuda et al., 2020, 2022). In conclusion, a comprehensive understanding of the sex determination mechanism will not only shed light on the repeated evolution of dioecy in the plant kingdom but also will promote the breeding application of sex regulatory genes in economically important dioecious plant species.

## Author contributions

XZ and WW: drafted the manuscript. XZ: original draft. LP, WG, YL, and WW: inputs and revision. All authors read and approved the submitted version.

## Funding

We acknowledge funding from the Rural Revitalization Project of Guangdong Province (KTP20210181) and the Innovation Project for Forestry Science and Technology in Guangdong (2021KJCX015).

## Conflict of interest

The authors declare that the research was conducted in the absence of any commercial or financial relationships that could be construed as a potential conflict of interest.

## Publisher's note

All claims expressed in this article are solely those of the authors and do not necessarily represent those of their affiliated organizations, or those of the publisher, the editors and the reviewers. Any product that may be evaluated in this article, or claim that may be made by its manufacturer, is not guaranteed or endorsed by the publisher.
