# Supplementary material for: A convergent mechanism of sex determination in dioecious plants: Distinct sex-determining genes display converged regulation on floral B-class genes
Source: Front Plant Sci. 2022 Aug 26;13:953445. doi: 10.3389/fpls.2022.953445 (PMC9459113; doi:10.3389/fpls.2022.953445)
Supplement: Supplementary file 1 [file Table_1.docx]

**Supplementary Table S1.** Dioecious plants with reference genome and convinced sexual system in a phylogenetic perspective

| **Family** | **Species** | **Sexual system** | | **Plastome record** | **Reference** |
| --- | --- | --- | --- | --- | --- |
|  |  | **Female** | **Male** |  |  |
| Ditrichaceae | *Ceratodon purpureus* | U | V | PRJNA258984 | [1] |
| Marchantiaeceae | *Marchantia inflexa* | U | V | PRJNA476339 | [2] |
|  | *Marchantia polymorpha* | U | V | NC_001319.1 | [3] |
| Cycadaceae | *Cycas panzhihuaensis* | XX | XY | NC_031413.1 | [4] |
| Ginkoaceae | *Ginkgo biloba* | XX | XY | NC_027976.1 | [5,6] |
| Amborellaceae | *Amborella trichopoda* | ZW | ZZ | NC_005086.1 | [7] |
| Asparagaceae | *Asparagus officinalis* | XX | XY | NC_034777.1 | [8,9] |
| Arecaceae | *Phoenix dactylifera* | XX | XY | NC_013991.2 | [10] |
| Dioscoreaceae | *Dioscorea alata* | XX | XY | NC_039707.1 | [11] |
|  | *Dioscorea rotundata* | ZW | ZZ | NC_024170.1 | [12] |
| Actinidiaceae | *Actinidia chinensis* | XX | XY | NC_026690.1 | [13,14] |
| Ebenaceae | *Diospyros lotus* | XX | XY | NC_030786.1 | [15,16] |
| Amaranthaceae | *Spinacia oleracea* | XX | XY | NC_002202.1 | [17] |
|  | *Amaranthus tuberculatus* | XX | XY | PRJNA38543 | [18] |
| Polygonaceae | *Rumex hastatulus* | XX | XY | NC_050928.1 | [19] |
| Vitaceae | *Vitis vinifera* | XX | XY | NC_007957.1 | [20] |
| Caricaceae | *Carica papaya* | XX | XY | NC_010323.1 | [21,22] |
| Anacardiaceae | *Pistacia vera* | ZW | ZZ | NC_034998.1 | [23] |
| Myricaceae | *Morella rubra* | ZW | ZZ | NC_035006.1 | [24] |
| Rosaceae | *Fragaria × ananassa* | ZW | ZZ | NC_035961.1 | [25,26] |
| Elaeagnaceae | *Hippophae rhamnoides* | XX | XY | NC_035548.1 | [27] |
| Moraceae | *Ficus hispida* | XX | XY | NC_053834.1 | [28] |
| Cannabaceae | *Cannabis sativa* | XX | XY | NC_027223.1 | [29] |
|  | *Humulus lupulus* | XX | XY | NC_028032.1 | [30] |
| Euphorbiaceae | *Mercurialis annua* | XX | XY | PRJNA369310 | [31] |
| Salicaceae | *Populus alba* | ZW | ZZ | NC_008235.1 | [32] |
|  | *Populus deltoides* | XX | XY | NC_040929.1 | [33] |
|  | *Populus euphratica* | XX | XY | NC_024747.1 | [34] |
|  | *Populus tremula* | XX | XY | NC_027425.1 | [32] |
|  | *Populus trichocarpa* | XX | XY | NC_009143.1 | [32,35] |
|  | *Salix purpurea* | ZW | ZZ | NC_029693.1 | [36] |
|  | *Salix suchowensis* | ZW | ZZ | NC_026462.1 | [37] |
|  | *Salix viminalis* | ZW | ZZ | PRJEB31619 | [38] |

**Reference**

1. Carey, S.B.; Jenkins, J.; Lovell, J.T.; Maumus, F.; Sreedasyam, A.; Payton, A.C.; Shu, S.; Tiley, G.P.; Fernandez-Pozo, N.; Barry, K. The *Ceratodon purpureus* genome uncovers structurally complex, gene rich sex chromosomes. *bioRxiv* **2020**, 163634.

2. Marks, R.A.; Smith, J.J.; Cronk, Q.; Grassa, C.J.; McLetchie, D.N. Genome of the tropical plant *Marchantia inflexa*: implications for sex chromosome evolution and dehydration tolerance. *Scientific reports* **2019**, *9*, 1-13.

3. Bowman, J.L.; Kohchi, T.; Yamato, K.T.; Jenkins, J.; Shu, S.; Ishizaki, K.; Yamaoka, S.; Nishihama, R.; Nakamura, Y.; Berger, F. Insights into land plant evolution garnered from the *Marchantia polymorpha* genome. *Cell* **2017**, *171*, 287-304. e215.

4. Liu, Y.; Wang, S.; Li, L.; Yang, T.; Dong, S.; Wei, T.; Wu, S.; Liu, Y.; Gong, Y.; Feng, X. The *Cycas* genome and the early evolution of seed plants. *Nature Plants* **2022**, 1-13.

5. Liao, Q.; Du, R.; Gou, J.; Guo, L.; Shen, H.; Liu, H.; Nguyen, J.K.; Ming, R.; Yin, T.; Huang, S. The genomic architecture of the sex‐determining region and sex‐related metabolic variation in *Ginkgo biloba*. *The Plant Journal* **2020**, *104*, 1399-1409.

6. Zhang, H.; Zhang, R.; Yang, X.; Gu, K.-J.; Chen, W.; Chang, Y.; Xu, Q.; Liu, Q.; Qin, Y.; Hong, X. Recent origin of an XX/XY sex-determination system in the ancient plant lineage *Ginkgo biloba*. *bioRxiv* **2019**, 517946.

7. Käfer, J.; Bewick, A.; Andres‐Robin, A.; Lapetoule, G.; Harkess, A.; Caïus, J.; Fogliani, B.; Gâteblé, G.; Ralph, P.; dePamphilis, C.W. A derived ZW chromosome system in *Amborella trichopoda*, representing the sister lineage to all other extant flowering plants. *New Phytologist* **2022**, *233*, 1636-1642.

8. Harkess, A.; Huang, K.; van der Hulst, R.; Tissen, B.; Caplan, J.L.; Koppula, A.; Batish, M.; Meyers, B.C.; Leebens-Mack, J. Sex determination by two Y-linked genes in garden asparagus. *The Plant Cell* **2020**, *32*, 1790-1796.

9. Harkess, A.; Zhou, J.; Xu, C.; Bowers, J.E.; Van der Hulst, R.; Ayyampalayam, S.; Mercati, F.; Riccardi, P.; McKain, M.R.; Kakrana, A. The asparagus genome sheds light on the origin and evolution of a young Y chromosome. *Nature Communications* **2017**, *8*, 1279.

10. Torres, M.F.; Mathew, L.S.; Ahmed, I.; Al-Azwani, I.K.; Krueger, R.; Rivera-Nuñez, D.; Mohamoud, Y.A.; Clark, A.G.; Suhre, K.; Malek, J.A. Genus-wide sequencing supports a two-locus model for sex-determination in *Phoenix*. *Nature Communications* **2018**, *9*, 1-9.

11. Cormier, F.; Lawac, F.; Maledon, E.; Gravillon, M.-C.; Nudol, E.; Mournet, P.; Vignes, H.; Arnau, G. A reference high-density genetic map of greater yam (*Dioscorea alata* L.). *Theoretical and Applied Genetics* **2019**, *132*, 1733-1744.

12. Tamiru, M.; Natsume, S.; Takagi, H.; White, B.; Yaegashi, H.; Shimizu, M.; Yoshida, K.; Uemura, A.; Oikawa, K.; Abe, A. Genome sequencing of the staple food crop white Guinea yam enables the development of a molecular marker for sex determination. *BMC Biology* **2017**, *15*, 1-20.

13. Akagi, T.; Henry, I.M.; Ohtani, H.; Morimoto, T.; Beppu, K.; Kataoka, I.; Tao, R. A Y-encoded suppressor of feminization arose via lineage-specific duplication of a cytokinin response regulator in kiwifruit. *The Plant Cell* **2018**, *30*, 780-795.

14. Akagi, T.; Pilkington, S.M.; Varkonyi-Gasic, E.; Henry, I.M.; Sugano, S.S.; Sonoda, M.; Firl, A.; McNeilage, M.A.; Douglas, M.J.; Wang, T. Two Y-chromosome-encoded genes determine sex in kiwifruit. *Nature Plants* **2019**, *5*, 801-809.

15. Akagi, T.; Henry, I.M.; Tao, R.; Comai, L. A Y-chromosome–encoded small RNA acts as a sex determinant in persimmons. *Science* **2014**, *346*, 646-650.

16. Akagi, T.; Shirasawa, K.; Nagasaki, H.; Hirakawa, H.; Tao, R.; Comai, L.; Henry, I.M. The persimmon genome reveals clues to the evolution of a lineage-specific sex determination system in plants. *PLoS Genetics* **2020**, *16*, e1008566.

17. Ma, X.; Yu, L.a.; Fatima, M.; Wadlington, W.H.; Hulse-Kemp, A.M.; Zhang, X.; Zhang, S.; Xu, X.; Wang, J.; Huang, H. The spinach YY genome reveals sex chromosome evolution, domestication, and introgression history of the species. *Genome Biology* **2022**, *23*, 1-30.

18. Montgomery, J.S.; Giacomini, D.A.; Weigel, D.; Tranel, P.J. Male‐specific Y‐chromosomal regions in waterhemp (*Amaranthus tuberculatus*) and Palmer amaranth (*Amaranthus palmeri*). *New Phytologist* **2021**, *229*, 3522-3533.

19. Rifkin, J.L.; Beaudry, F.E.; Humphries, Z.; Choudhury, B.I.; Barrett, S.C.; Wright, S.I. Widespread recombination suppression facilitates plant sex chromosome evolution. *Molecular Biology and Evolution* **2021**, *38*, 1018-1030.

20. Massonnet, M.; Cochetel, N.; Minio, A.; Vondras, A.M.; Lin, J.; Muyle, A.; Garcia, J.F.; Zhou, Y.; Delledonne, M.; Riaz, S. The genetic basis of sex determination in grapes. *Nature Communications* **2020**, *11*, 1-12.

21. Liu, Z.; Moore, P.H.; Ma, H.; Ackerman, C.M.; Ragiba, M.; Yu, Q.; Pearl, H.M.; Kim, M.S.; Charlton, J.W.; Stiles, J.I. A primitive Y chromosome in papaya marks incipient sex chromosome evolution. *Nature* **2004**, *427*, 348.

22. Wang, J.; Na, J.-K.; Yu, Q.; Gschwend, A.R.; Han, J.; Zeng, F.; Aryal, R.; VanBuren, R.; Murray, J.E.; Zhang, W. Sequencing papaya X and Yh chromosomes reveals molecular basis of incipient sex chromosome evolution. *Proceedings of the National Academy of Sciences* **2012**, *109*, 13710-13715.

23. Zeng, L.; Tu, X.-L.; Dai, H.; Han, F.-M.; Lu, B.-S.; Wang, M.-S.; Nanaei, H.A.; Tajabadipour, A.; Mansouri, M.; Li, X.-L. Whole genomes and transcriptomes reveal adaptation and domestication of pistachio. *Genome Biology* **2019**, *20*, 1-13.

24. Jia, H.M.; Jia, H.J.; Cai, Q.L.; Wang, Y.; Zhao, H.B.; Yang, W.F.; Wang, G.Y.; Li, Y.H.; Zhan, D.L.; Shen, Y.T. The red bayberry genome and genetic basis of sex determination. *Plant Biotechnology Journal* **2019**, *17*, 397-409.

25. Edger, P.P.; Poorten, T.J.; VanBuren, R.; Hardigan, M.A.; Colle, M.; McKain, M.R.; Smith, R.D.; Teresi, S.J.; Nelson, A.D.; Wai, C.M. Origin and evolution of the octoploid strawberry genome. *Nature Genetics* **2019**, *51*, 541-547.

26. Tennessen, J.A.; Wei, N.; Straub, S.C.; Govindarajulu, R.; Liston, A.; Ashman, T.-L. Repeated translocation of a gene cassette drives sex-chromosome turnover in strawberries. *PLoS Biology* **2018**, *16*, e2006062.

27. Wu, Z.; Chen, H.; Pan, Y.; Feng, H.; Fang, D.; Yang, J.; Wang, Y.; Yang, J.; Sahu, S.K.; Liu, J. Genome of *Hippophae rhamnoides* provides insights into a conserved molecular mechanism in actinorhizal and rhizobial symbioses. *New Phytologist* **2022**.

28. Zhang, X.; Wang, G.; Zhang, S.; Chen, S.; Wang, Y.; Wen, P.; Ma, X.; Shi, Y.; Qi, R.; Yang, Y. Genomes of the banyan tree and pollinator wasp provide insights into fig-wasp coevolution. *Cell* **2020**, *183*, 875-889. e817.

29. Gao, S.; Wang, B.; Xie, S.; Xu, X.; Zhang, J.; Pei, L.; Yu, Y.; Yang, W.; Zhang, Y. A high-quality reference genome of wild *Cannabis sativa*. *Horticulture Research* **2020**, *7*.

30. Padgitt-Cobb, L.K.; Kingan, S.B.; Wells, J.; Elser, J.; Kronmiller, B.; Moore, D.; Concepcion, G.; Peluso, P.; Rank, D.; Jaiswal, P. A phased, diploid assembly of the Cascade hop (*Humulus lupulus*) genome reveals patterns of selection and haplotype variation. *BioRxiv* **2019**, 786145.

31. Veltsos, P.; Ridout, K.E.; Toups, M.A.; González-Martínez, S.C.; Muyle, A.; Emery, O.; Rastas, P.; Hudzieczek, V.; Hobza, R.; Vyskot, B. Early sex-chromosome evolution in the diploid dioecious plant *Mercurialis annua*. *Genetics* **2019**, *212*, 815-835.

32. Müller, N.A.; Kersten, B.; Leite Montalvão, A.P.; Mähler, N.; Bernhardsson, C.; Bräutigam, K.; Carracedo Lorenzo, Z.; Hoenicka, H.; Kumar, V.; Mader, M. A single gene underlies the dynamic evolution of poplar sex determination. *Nature Plants* **2020**, *6*, 630-637.

33. Xue, L.; Wu, H.; Chen, Y.; Li, X.; Hou, J.; Lu, J.; Wei, S.; Dai, X.; Olson, M.S.; Liu, J. Evidences for a role of two Y-specific genes in sex determination in *Populus deltoides*. *Nature Communications* **2020**, *11*, 1-12.

34. Yang, W.; Wang, D.; Li, Y.; Zhang, Z.; Tong, S.; Li, M.; Zhang, X.; Zhang, L.; Ren, L.; Ma, X. A general model to explain repeated turnovers of sex determination in the Salicaceae. *Molecular Biology and Evolution* **2021**, *38*, 968-980.

35. Hofmeister, B.T.; Denkena, J.; Colomé-Tatché, M.; Shahryary, Y.; Hazarika, R.; Grimwood, J.; Mamidi, S.; Jenkins, J.; Grabowski, P.P.; Sreedasyam, A. A genome assembly and the somatic genetic and epigenetic mutation rate in a wild long-lived perennial *Populus trichocarpa*. *Genome Biology* **2020**, *21*, 1-27.

36. Zhou, R.; Macaya-Sanz, D.; Carlson, C.H.; Schmutz, J.; Jenkins, J.W.; Kudrna, D.; Sharma, A.; Sandor, L.; Shu, S.; Barry, K. A willow sex chromosome reveals convergent evolution of complex palindromic repeats. *Genome Biology* **2020**, *21*, 1-19.

37. Dai, X.; Hu, Q.; Cai, Q.; Feng, K.; Ye, N.; Tuskan, G.A.; Milne, R.; Chen, Y.; Wan, Z.; Wang, Z. The willow genome and divergent evolution from poplar after the common genome duplication. *Cell Research* **2014**, *24*, 1274-1277.

38. Almeida, P.; Proux-Wera, E.; Churcher, A.; Soler, L.; Dainat, J.; Pucholt, P.; Nordlund, J.; Martin, T.; Rönnberg-Wästljung, A.-C.; Nystedt, B. Genome assembly of the basket willow, *Salix viminalis*, reveals earliest stages of sex chromosome expansion. *BMC Biology* **2020**, *18*, 1-18.
